# Functional status of microvascular vasomotion is impaired in spontaneously hypertensive rat

**DOI:** 10.1038/s41598-017-17013-w

**Published:** 2017-12-06

**Authors:** Mingming Liu, Xiaoyan Zhang, Bing Wang, Qingbin Wu, Bingwei Li, Ailing Li, Honggang Zhang, Ruijuan Xiu

**Affiliations:** Institute of Microcirculation, Key Laboratory of Microcirculation, Ministry of Health, Chinese Academy of Medical Sciences & Peking Union Medical College, Beijing, 100005 China

## Abstract

Accumulating evidence demonstrates that microcirculation plays a role in the pathogenesis of hypertension. In the current study, we demonstrated that pancreatic islet microvascular vasomotion of spontaneously hypertensive rats (SHRs) lost the ability to regulate blood flow perfusion and exhibited a lower microvascular blood perfusion pattern which was negative correlated with blood glucose level. SHRs administrated with insulin revealed an improvement of pancreatic islet microvascular vasomotion and blood perfusion pattern. *In vitro*, the expressions of endothelial nitric oxide synthase (eNOS) and phospho-eNOS^ser1177^ (p-eNOS^ser1177^) were significantly decreased in high glucose exposed islet endothelial cells (iECs), accompanied with a higher ratio of eNOS monomer to eNOS dimer and a significantly increased malondialdehyde and nitrite levels. Meanwhile, barrier function, tube formation and migration capacities of high glucose exposed iECs were significantly inhibited. In contrast, iECs dysfunction induced by glucose toxicity and oxidative stress was attenuated or improved by supplement with insulin, L-arginine and β-mercaptoethanol. In summary, our findings suggest that functional status of pancreatic islet microvascular vasomotion is impaired in SHRs and provide evidence that treatment with insulin, L-arginine and β-mercaptoethanol improves endothelium-dependent microvascular vasomotion and meliorates iECs function due to anti-hyperglycemic and anti-oxidative effects, partly through mechanism involving regulation of eNOS and p-eNOS^ser1177^.

## Introduction

Hypertension is one of the most common chronic disorder that is considered a crucial risk factor for vascular diseases^1^. Recent emerging evidence derived from experimental animals and clinical patients has indicated that the small arteries and arterioles response for the majority of vascular resistance to blood flow^2^ and the regulation of flow resistance is inadequate in hypertension^3^. Microcirculation includes arterioles, capillaries and venules and is responsible for optimizing nutrient and oxygen supply to meet physiological demand and preventing fluctuations in hydrostatic pressure. It is widely acknowledged that microcirculation serves to protect capillaries from potential adverse effects of high perfusion pressure. Capillary rarefaction and the concomitant resistance artery remodeling increase peripheral resistance and capillaries stiffness occurring in hypertensive individuals increases their central systolic and pulse pressures^4^. Therefore, microcirculation is extremely important for maintaining peripheral resistance and blood pressure within the normal ranges.

Microvascular vasomotion, defined as the rhythmic dilatation and contraction of microvessels, is a hemodynamic parameter that is generally used to represent the functional status of microcirculation^5^. Physiological microvascular oscillation regulates the microvascular blood flow distribution and maintains the normal function of local organs and tissues, whereas dysfunction of microvascular vasomotion impedes arterial and capillary blood perfusion and oxygenation, thus contributing to detrimental hemodynamic effects of blood pressure to organs^6^. The importance of microvascular disturbance in hypertensive lesions of organs has been noticed for several years^7^, and the accumulating data demonstrates that there should be a link between microvascular vasomotion and hypertension. Early changes in the microvasculature in hypertensive individuals are characterized by abnormal blood perfusion of microvessels. In a 10-year follow-up study, it was proved that the outcome of hypertensive patients was associated with microcirculatory functional status^8^. As chronic hypertension possibly leads to microcirculatory pathological changes as mentioned above, the notion that microcirculation contributes to hypertension pathogenesis is reasonable^9^. Identifying the linkage between microcirculatory dysfunction and hypertension may provide a new possible explanation for the pathogenesis of hypertension. However, the mechanisms behind these microcirculatory pathological phenomena have been debated for years, which are remained incompletely clarified.

It should be noted that hypertension may be only one symptom among a larger set of metabolic concurrent phenomena. A higher prevalence of hyperglycemia and higher fasting plasma glucose within the normal range were observed among clinical individuals with hypertension^10^. In a multistage and stratified epidemiological survey reported by the China National Diabetes and Metabolic Disorders Study Group, it was reported that severe hypertension was associated with impaired glucose tolerance^11^. Due to pancreatic islet is responsible for the regulation of blood glucose level, we hypothesize that metabolic abnormality (such as hyperglycemia) may get involved in microcirculatory dysfunction and contribute to the injury of microvascular endothelial cells. In the current study, we investigated the functional status of pancreatic islet microvascular vasomotion in spontaneously hypertensive rats (SHRs) and its normotensive control Wistar Kyoto rats (WKYs) using laser Doppler and analyzed microvascular blood perfusion pattern. Meanwhile, the role of glucose toxicity in islet endothelial cells (iECs) dysfunction was investigated.

## Results

### SHRs exhibited elevated blood glucose levels

The general characteristics of body weight, fasting plasma glucose and blood pressure of SHRs and normotensive control WKYs were shown in Table 1. Age and gender distribution were similar between the two groups, and no significant differences were found. The mean body weights of WKYs and SHRs were 171.7 g and 201.3 g, respectively. Systolic blood pressure (168.5 ± 2.1 mmHg vs 116.2 ± 2.5 mmHg), diastolic blood pressure (85.7 ± 2.7 mmHg vs 74.5 ± 3.1 mmHg) and mean arterial pressure (127.1 ± 2.0 mmHg vs 95.3 ± 2.3 mmHg) of SHRs were significantly higher than those of WKYs. Although the blood glucose level in both groups was lower than the diagnostic criteria for diabetes mellitus (200 mg/dL), the blood glucose level in SHRs (141.0 ± 2.3 mg/dL) was significantly higher than that in WKYs (106.5 ± 5.4 mg/dL).

**Table 1:** Body weight, blood glucose and blood pressure of WKYs and SHRs.

Groups	WKYs (*n* = 6)	SHRs (*n* = 6)
Age (weeks)	8	8
Gender (male/female)	6/0	6/0
Body weight (g)	171.7 ± 2.3	201.3 ± 4.3^**^
FPG (mg/dL)	106.5 ± 5.4	141.0 ± 2.3^**^
Blood pressure (mmHg)		
SBP	116.2 ± 2.5	168.5 ± 2.1^**^
DBP	74.5 ± 3.1	85.7 ± 2.7^*^
MAP	95.3 ± 2.3	127.1 ± 2.0^**^

Unless indicated otherwise, numbers were expressed as the mean ± S.E.M. (*n* = 6 in each group). FPG, fasting plasma glucose; SBP, systolic blood pressure; DBP, diastolic blood pressure; MAP, mean arterial pressure.*P < 0.05 compared with WKYs; **P < 0.01 compared with WKYs.

### Pancreatic islet microvascular vasomotion was abnormal in SHRs

**Figure 1:**
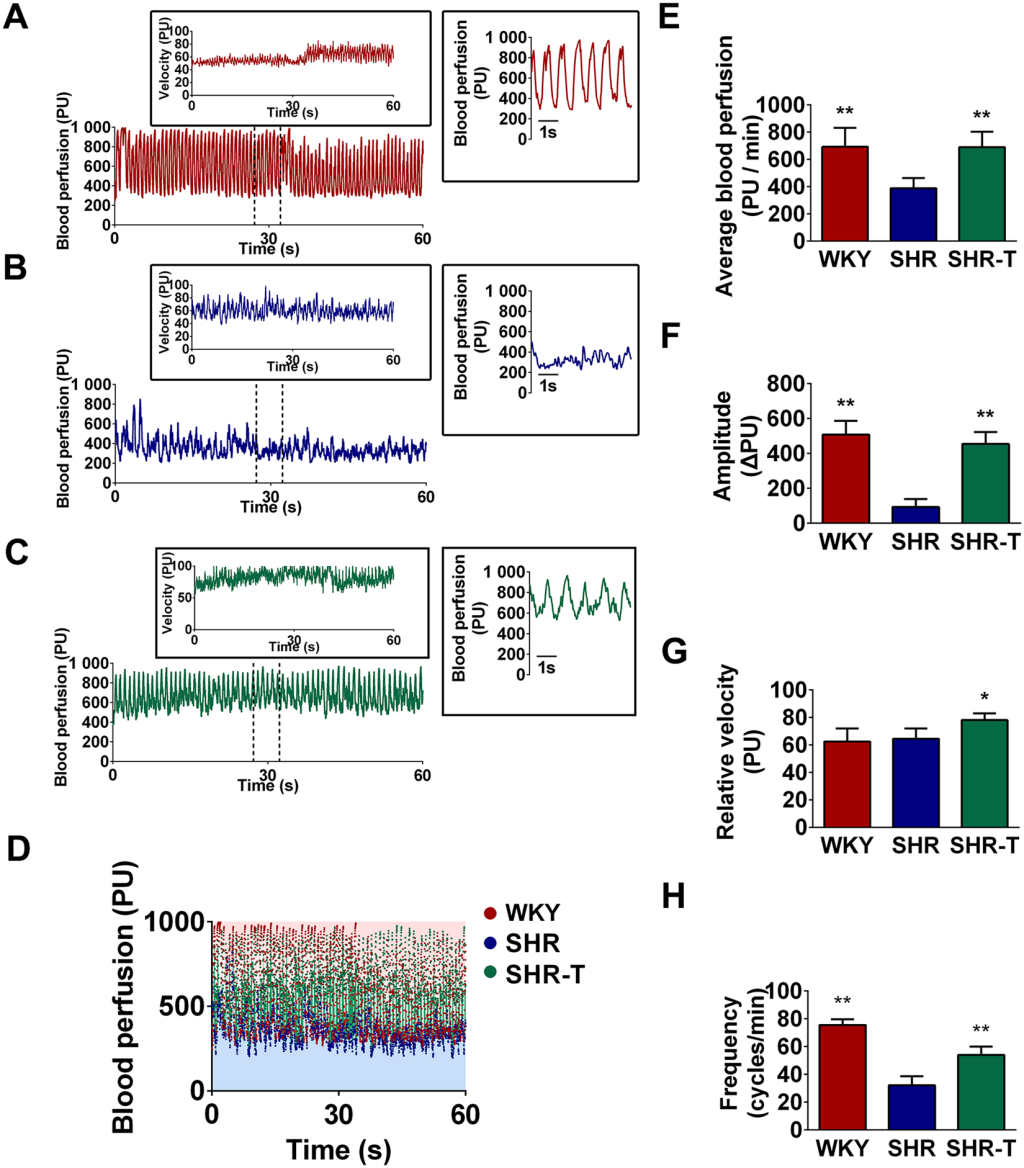
Functional status of pancreatic islet microvascular vasomotion was impaired in SHRs. (**A)** Functional status of pancreatic islet microvascular vasomotion in WKYs was assessed based on the dynamic microvascular blood perfusion data. **(B**) Functional status of pancreatic islet microvascular vasomotion in SHRs. **(C)** Functional status of pancreatic islet microvascular vasomotion in insulin treated SHRs (SHR-Ts). The dynamic velocity of microvascular blood flow was shown in the rectangular insert. The microvascular blood perfusion between the dashed lines (5 s) was extracted in the square insert. The red line represents microvascular blood perfusion data of WKYs; the blue line represents perfusion data of SHRs; the green line represents perfusion data of SHR-Ts; PU, perfusion unit. **(D**) Distribution patterns of pancreatic islet microvascular blood perfusion in WKYs, SHRs and SHR-Ts. The distribution pattern was compared using microvascular blood perfusion data derived from laser Doppler monitoring. WKYs and SHR-Ts showed a higher blood perfusion pattern, whereas SHRs displayed a relatively lower scale blood perfusion pattern. Red dots, blood perfusion of WKYs. Blue dots, blood perfusion of SHRs. Green dots, blood perfusion of SHR-Ts. PU, perfusion unit. **(E–H)** Quantification analysis of functional status of pancreatic islet microvascular vasomotion in WKYs, SHRs and SHR-Ts. **(E)** The average blood perfusion in SHRs was significantly lower than in WKYs and SHR-Ts. **(F)** The amplitude of the pancreatic islet microvascular vasomotion was significantly decreased in SHRs. **(G)** No significant difference in the relative velocity of pancreatic islet microvascular vasomotion was found between SHRs and WKYs. **(H)** The frequency of pancreatic islet microvascular vasomotion in SHRs was lower than in WKYs and SHR-Ts. *P < 0.05 compared with SHRs **P < 0.01 compared with SHRs.

In this study, our concern was that the functional status of pancreatic islet microvascular vasomotion should be impaired due to the microvascular endothelial cytotoxicity derived from elevated blood glucose in SHRs. We thus analyzed the distribution pattern of pancreatic islet of SHRs and WKYs using laser Doppler data. Meanwhile, considering that glucose toxicity may be one of the reasons that pancreatic islet microvascular vasomotion dysfunction, we administrated SHRs with 0.5 IU/day insulin (SHR-Ts) subcutaneously for one week to maintain blood glucose in normal level. SHR-Ts whose blood glucose level was 101.7 ± 7.0 mg/dL were subject to the same analysis. WKYs, SHRs and SHR-Ts generally showed different blood distribution and perfusion patterns of pancreatic islet microvascular vasomotion (Fig. 1D). Compared with hypertensive SHRs, a higher scale of blood perfusion of pancreatic islet microvascular vasomotion was observed in WKYs and SHR-Ts. The microcirculatory conditions of pancreatic islet were represented by the functional status of pancreatic islet microvascular vasomotion based on microvascular hemodynamic parameters, including average blood perfusion and the amplitude, frequency and relative velocity of microvascular blood flow. Our data showed that the rhythm of contractions and relaxations of pancreatic islet microvascular vasomotion was irregular in SHRs (Fig. 1B) compared to the normotensive control and insulin-treated rats (Fig. 1A,C). We then extracted the 5 s of data of pancreatic islet microvascular blood perfusion within the dash lines and demonstrated that the pancreatic islet microcirculation lost the ability to regulate the functional status of pancreatic islet microvascular vasomotion. Quantification analysis revealed a significantly decreased average blood perfusion (Fig. 1E) and the amplitude (Fig. 1F) and frequency (Fig. 1H) of pancreatic islet microvascular vasomotion in SHRs. However, no significantly difference was found in the relative velocity of pancreatic islet microvascular vasomotion among groups (Fig. 1G). The anti-hyperglycemic treatment led to a restoration of pancreatic islet microvascular vasomotion and the distribution pattern of SHRs (Fig. 1C–H). Furthermore, we assessed the pancreatic islet microvascular vasomotion of a group of three-week-old SHRs due to the blood glucose had not elevated yet in the early weeks. The general characteristics of three-week-old WKYs and SHRs were listed in Supplementary Table S1. Both three-week-old rats revealed a parallel blood distribution and perfusion pattern and no significant differences were found in functional status of pancreatic islet microvascular vasomotion (Supplementary Fig. S1). Taken together, these data indicated that glucose toxicity may play a role in the pathogenesis of pancreatic islet microvascular vasomotion impairment.

### Function of pancreatic islets was impaired in SHRs

**Figure 2:**
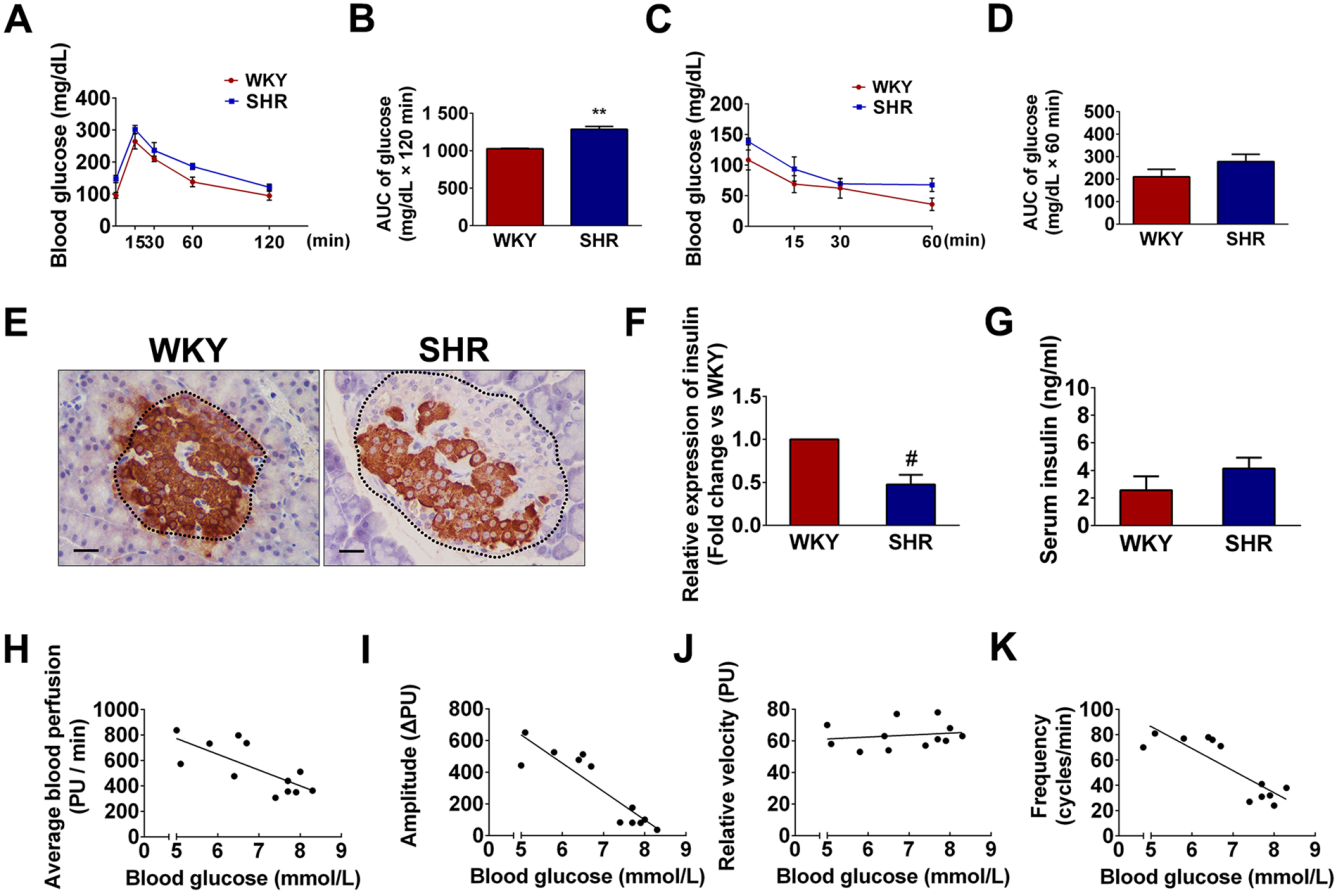
Impaired pancreatic islet function in SHRs was negatively correlated with functional status of pancreatic islet microvascular vasomotion. (**A**) Intraperitoneal glucose tolerance test. Fasted rats in both groups received an *i.p*. injection of glucose (2 g/kg body weight). Blood glucose levels were measured at the indicated times (0, 15, 30, 60 and 120 min). SHRs exhibited significantly impaired glucose tolerance following glucose administration compared with its normotensive WKYs. (**B**) Area under the curve (AUC) was calculated by the trapezoidal method to measure the degree of blood glucose tolerance impairment. SHRs had higher AUC_glucose_ values compared with WKYs. (**C**) Insulin tolerance test. Non-fasted rats in both groups were *i.p*. administered insulin (1 IU/kg body weight), and blood glucose levels were measured as above at the indicated times (0, 15, 30 and 60 min). (**D**) AUC_insulin_ was calculated by the trapezoidal method mentioned above, and the AUC_insulin_ of WKYs tended to be lower than that of SHRs, although the difference was not significant. (●) represents blood glucose levels of WKYs and (■) represents blood glucose levels of SHRs. Data are presented as mean ± S.E.M. (*n* = 6 per group). **P < 0.01 compared with WKYs. (**E**) The expressions of insulin in pancreatic islets were revealed by immunohistochemical staining. Representative islets were circled by dashed line. Scale bar = 20 μm. (**F**) The optical density value was normalized to WKYs and relative expression of insulin was compared. ^#^ *P* < 0.01 compared with WKYs. (**G**) Serum insulin levels of WKYs and SHRs were measured by ELISA. No significant difference was found between rats. (**H**–**K**) Correlations between blood glucose level and functional status of pancreatic islet microvascular vasomotion. Negative correlations were found between blood glucose level and (**H**) average blood perfusion (*r* = −0.7369, *P* = 0.0063), (**I**) amplitude (*r* = −0.9035, *P* = 0.0001), and (**K**) frequency (*r* = −0.8554, *P* = 0.0004). (**J**) No correlation was found between blood glucose level and the relative velocity of pancreatic islet microvascular vasomotion. PU, perfusion unit.

Intraperitoneal glucose tolerance test and insulin tolerance test were performed to determine pancreatic islet β cells function in SHRs and WKYs. As shown in Fig. 2A, blood glucose levels over the entire time course of the glucose tolerance test were higher in SHRs than in WKYs, while the SHRs had a higher AUC_glucose_ than the normotensive control rats (Fig. 2B). These results suggested that SHRs had impaired glucose tolerance following glucose injection compared with WKYs. In response to insulin administration, WKYs showed a greater and continuing decrease in blood glucose levels while the reduction of blood glucose in SHRs exhibited a plateau (Fig. 2C). In addition, the baseline blood glucose level was significantly lower in WKYs than in SHRs. No significantly difference in the AUC_insulin_ was found between WKYs and SHRs (Fig. 2D). In addition, immunochemical staining revealed that insulin level in pancreatic islets was significantly decreased in SHRs compared with WKYs (Fig. 2E,F), whereas serum insulin level was comparable between two groups (Fig. 2G).

### Correlations between blood glucose level and pancreatic islet microvascular vasomotion parameters

Our previous study suggests that pancreatic islet microvascular vasomotion plays a key role in regulating blood glucose level and maintaining physiological function of pancreatic islet^12^. Therefore, we analyzed the correlation between blood glucose level and pancreatic islet microvascular vasomotion and found that average blood perfusion (*r* = −0.7369, *P* < 0.01) and the amplitude (*r* = −0.9035, *P* < 0.01) and frequency (*r* = −0.8554, *P* < 0.01) of pancreatic islet microvascular vasomotion were negatively correlated with blood glucose level (Fig. 2H,I and K). However, the relative velocity (*r* = 0.1704, *P* > 0.05) of pancreatic islet microvascular vasomotion was not significantly correlated with blood glucose levels (Fig. 2J), suggesting that elevated blood glucose may not the only determinant in the deteriorating of pancreatic islet microvascular vasomotion. These findings indicate that elevated glucose level might lead to the deterioration of pancreatic islet microvascular vasomotion in SHRs.

### Dysfunction of high glucose exposed iECs

**Figure 3:**
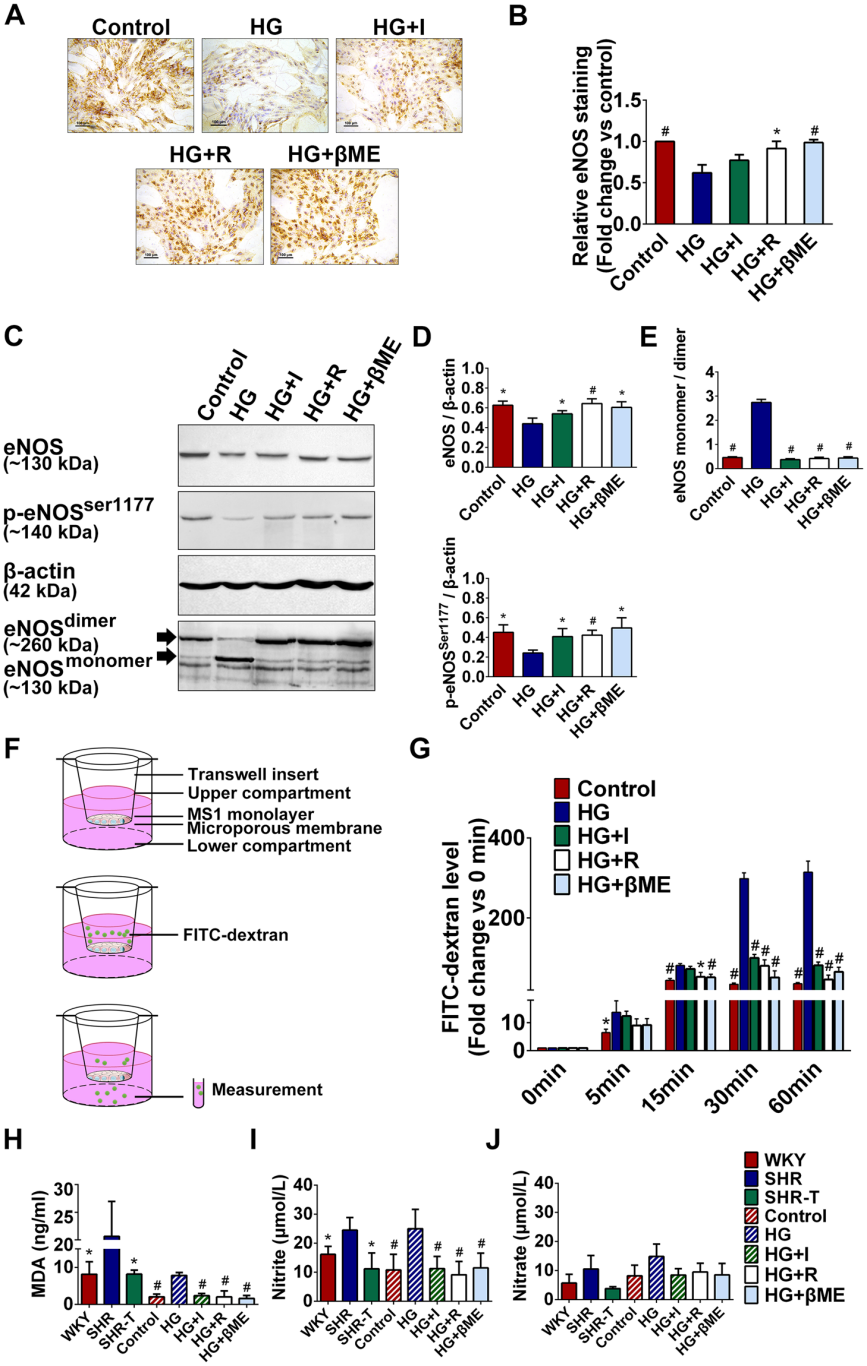
Glucose toxicity impaired iECs function. (**A**) MS1 were treated with 5.6 mM glucose (control group), 35 mM glucose (HG group), 35 mM glucose plus 10^−8^ M insulin (HG + I group), 35 mM glucose plus 0.5 mM L-arginine (HG + R group) and 35 mM glucose plus 100 μM β-mercaptoethanol (HG + βME group) respectively and the expressions of eNOS on MS1 were revealed by immune-labelling. Scale bar = 100 μm. **(B)** Quantitation of immunohistochemical analysis of eNOS expression. The expression of eNOS was normalized against control MS1, relative eNOS staining of other groups were calculated. **(C,D)** The expression levels of eNOS and p-eNOS^ser1177^ in different groups were analyzed by Western blotting. And the same protein samples were subjected to low temperature SDS-PAGE to evaluate eNOS dimer and monomer. Representative Western blotting images were cropped from the full-length blots. The full-length Western blots were included in the Supplementary information. *P < 0.05 compared with HG group **P < 0.01 compared with HG group. (**E**) The densitometric ratio of eNOS monomer and dimer. ^#^ *P* < 0.01 compared with HG group. **(F)** A schematic diagram of Transwell-based permeability assay. MS1 were seeded at full confluence on a microporous membrane in the upper chamber. After treatment for 24 h by conditioned medium as mentioned above, 2 mg/ml FITC-conjugated dextran was added to the upper chamber. The passage of FITC-dextran was collected from the lower chamber at 0, 5, 15, 30 and 60 min and measured. **(G)** The fluorescence value of leaked FITC-dextran was normalized against the baseline (0 min) respectively. *P < 0.05 compared with HG group, ^#^ *P* < 0.01 compared with HG group. **(H)** MDA levels in plasma of rats and in cell culture supernatants of MS1. *P < 0.05 compared with SHR group, ^#^ *P* < 0.01 compared with HG group. **(I)** Nitrite levels in plasma of rats and in cell culture supernatants of MS1. *P < 0.05 compared with SHR group, ^#^ *P* < 0.05 compared with HG group. **(J)** The nitrate levels in plasma of rats and in cell culture supernatants of MS1.

Generally, endothelial dysfunction is characterized by an increase in microvascular permeability which is associated with endothelial nitric oxide synthase (eNOS). Based on the microvascular disturbance was observed in hyperglycemic SHRs, we therefore queried whether the eNOS expression of iECs was abnormal in the occurrence of high glucose *in vitro*. Immunostaining analysis revealed that the distributions of eNOS on MS1 under different glucose concentrations (Fig. 3A). Quantitative analysis showed that the expression of eNOS level was 0.6-fold decreased in 35 mM glucose exposed group (HG group) compared with control group (5.6 mM glucose), while insulin treated MS1 (35 mM glucose plus 10^−8^ M insulin, HG + I group) exhibited a trend that insulin restored the expression of eNOS on iECs, which did not reach the statistically significant threshold. In addition, MS1 treated with L-arginine (35 mM glucose plus 0.5 mM L-arginine, HG + R group) and β-mercaptoethanol (35 mM glucose plus 100 μM β-mercaptoethanol, HG + βME group) showed a significant increase of eNOS level (Fig. 3B). Western blotting demonstrated that the presences of total eNOS and phosphorylated eNOS (p-eNOS^ser1177^) were decreased in high glucose exposed MS1 (Fig. 3C,D). Meanwhile, high glucose exposed MS1 exhibited a significantly higher ratio of eNOS monomer to eNOS dimer, which was significantly altered by supplement with insulin, L-arginine and β-mercaptoethanol respectively (Fig. 3C,E).

It has been demonstrated that eNOS is generally involved in the endothelium-mediated relaxation and the maintenance of barrier function of endothelial cells. Therefore, our next experiment was designed to assess whether the permeability was abnormal and to evaluate the integrity of high glucose exposed MS1 monolayer *in vitro*. MS1 cells were seeded onto microporous membrane filter and fluorescein isothiocyanate (FITC)-dextran (a macromolecule with a size larger than 3 nm) was added into upper chamber of the Transwell system. The degree of FITC-dextran permeability was then determined by measuring FITC-dextran in the lower chambers using fluorometry (Fig. 3F). FITC-dextran level in lower chamber of the HG group was significantly higher than that of the control group whose fluorescent level in lower chamber was progressively increased (Fig. 3G). In addition, the increase of fluorescent levels at 30 min and 60 min time-points were significantly inhibited in insulin-treated, L-arginine- and β-mercaptoethanol-supplemented MS1, whose FITC-dextran level remained comparable to that of the control MS1 (Fig. 3G).

Furthermore, SHRs had significantly higher plasma malondialdehyde (MDA) level (Fig. 3H) and endogenous nitrite level (Fig. 3I) compared to WKYs. The decreases of MDA and nitrite levels were observed in insulin treated SHRs (SHR-Ts). Similarly, *in vitro*, high glucose exposed MS1 also showed significantly increased MDA and nitrite levels. However, the increases of MDA and nitrite levels were notably attenuated by the addition of L-arginine and β-mercaptoethanol (Fig. 3H,I). The presence of high glucose did not significantly alter nitrate level both in SHRs and high glucose exposed MS1 compared with other groups (Fig. 3J).

### Angiogenic capacity was inhibited in high glucose exposed iECs

**Figure 4:**
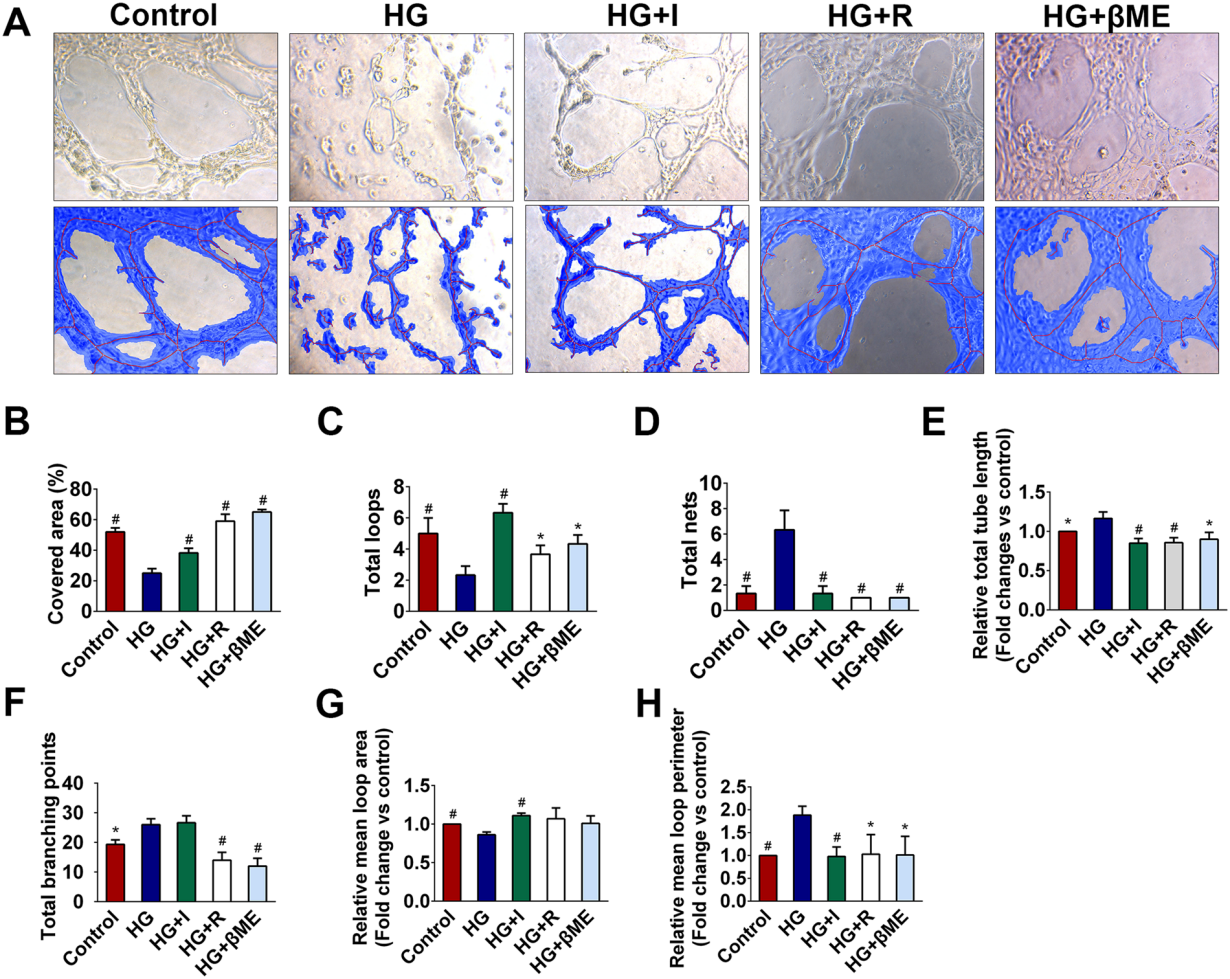
Glucose toxicity inhibited angiogenic capacity of iECs. **(A)** Capillary-like tubular structures of MS1 on Matrigel after treatment with 5.6 mM glucose (control), 35 mM glucose (HG group), 35 mM glucose plus 10^−8^ M insulin (HG + I group), 35 mM glucose plus 0.5 mM L-arginine (HG + R group) and 35 mM glucose plus 100 μM β-mercaptoethanol (HG + βME group) for 24 h (upper panel). Tubes and nets were automatically selected and marked in blue using Wimasis Image Analysis System (lower panel). Blue, the tubular structure. Red, tubes. White, branching points. **(B–H)** Quantitative analysis of tube formation capacity among groups. **(B,C)** The covered area (%) and total loop numbers were significantly decreased in HG group. **(D,E)** Total nets and relative total tube length (normalized against control group) in HG group were significantly higher than those in other groups. **(F)** Total branching points of capillary-like tubular structures of MS1 from control, HG, HG + I, HG + R and HG + βME group. **(G)** Relative mean loop area (normalized against control group) of MS1 in different groups. **(H)** Relative mean loop perimeter (normalized against control group) of MS1 in different groups. *P < 0.05 compared with HG group, ^#^ *P* < 0.01 compared with HG group.

To evaluate the ability of angiogenesis, the tube formation assay was performed. After MS1 were seeded on Matrigel, capillary-like tubular structures were formed gradually, and some of the capillary-like tubes connected to each other created a mesh-like structure on the Matrigel (Fig. 4A, upper panel). The capillary-like tubular structure was formed more densely in control, HG + I, HG + R and HG + βME groups compared with HG group. Areas, tubes and nets were selected and analyzed (Fig. 4A, lower panel). Quantitative analysis of capillary-like tubular structures showed that the covered area (%) in HG group was ~50% decreased compared with control group. Whereas the covered area (%) of insulin-treated MS1 was increased 1.5-fold higher than HG group (Fig. 4B). Meanwhile, high glucose exposed MS1 showed the similar trend towards a decrease in the relative mean loop area (Fig. 4G). In addition, the number of tubes was significantly decreased when glucose toxicity occurrence in the media (Fig. 4C). However, due to there were unconnected fragmented nodes in HG group, the number of nets (Fig. 4D), relative total tube length (Fig. 4E) and total branching points (Fig. 4F) and relative mean loop perimeter (Fig. 4H) were significantly higher than in control group. And interestingly, it appeared that the tube formation capacity of high glucose exposed MS1 was restored after insulin, L-arginine and β-mercaptoethanol treatment (Fig. 4B–H), indicating that the effects of glucose toxicity on deteriorating angiogenic capacity of iECs.

### Effects of high glucose on migration capacity of iECs

**Figure 5:**
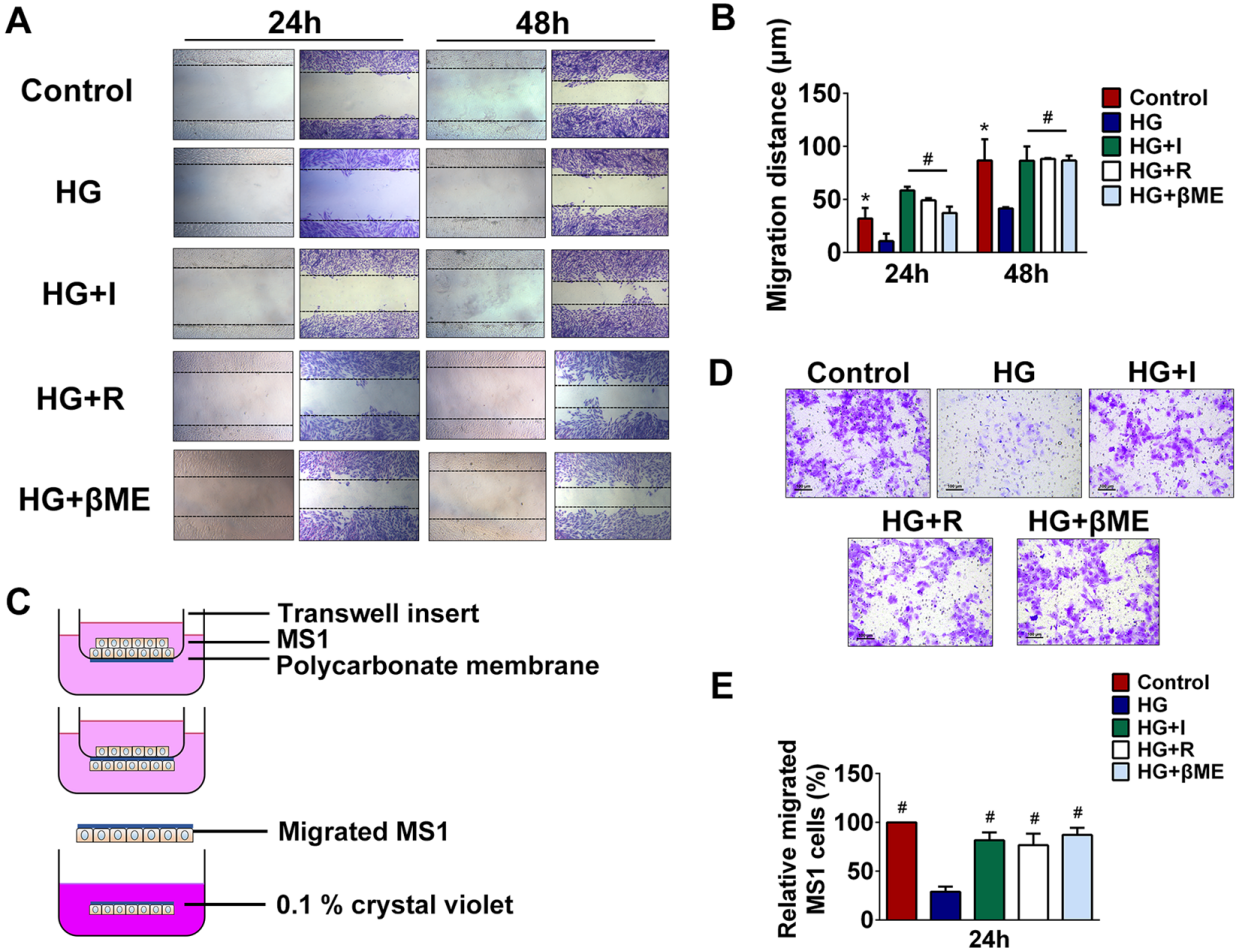
Glucose toxicity suppressed the migratory capacity of iECs. (**A)** MS1 cells were treated with 5.6 mM glucose (control), 35 mM glucose (HG group), 35 mM glucose plus 10^−8^ M insulin (HG + I group), 35 mM glucose plus 0.5 mM L-arginine (HG + R group) and 35 mM glucose plus 100 μM β-mercaptoethanol (HG + βME group) for 24 h or 48 h after scratching. **(B)** The migration distance in the wound healing assays were quantified according to the difference value between the covered area and the original wound area. *P < 0.05 compared with HG group, ^#^ *P* < 0.01 compared with HG group. (**C)** A schematic diagram of the Transwell-based glucotoxicity assay. MS1 were seeded on a polycarbonate membrane in the upper chamber and migrated MS1 were stained by 0.1% crystal violet. **(D)** MS1 were treated with 5.6 mM glucose (control), 35 mM glucose (HG group), 35 mM glucose plus 10^−8^ M insulin (HG + I group), 35 mM glucose plus 0.5 mM L-arginine (HG + R group) and 35 mM glucose plus 100 μM β-mercaptoethanol (HG + βME group) for 24 h and migrated MS1 were stained in violet. **(E)** The numbers of migrated MS1 were calculated in five different fields (200×) and relative migrated MS1 cells were compared with HG group. ^#^ *P* < 0.01 compared with HG group.

Evidence indicates that high glucose causes endothelial cells injury. We therefore designed using a wound healing assay to assess the effects of glucose toxicity on proliferation and migration capacities of iECs. Under the normal glucose concentration (5.6 mM), MS1 cells efficiently reduced the wound area in 48 h. Furthermore, the wound healing recovery was significantly inhibited both at 24 h and 48 h in HG group (Fig. 5A,B). In the presence of insulin, a significant increase of migration distance was observed in wound healing at 24 h and a restoration of wound healing was observed at 48 h (Fig. 5A,B). These results indicated that glucose toxicity could promote MS1 injury and destroy the migration capacity of iECs while insulin could protect MS1 from glucose toxicity-derived injury. Given the findings that oxidative stress may be involved in the iECs dysfunction derived from glucose toxicity, we evaluated the wound healing recovery in the presence of L-arginine and β-mercaptoethanol. Quantification of wound closure distances confirmed that nitric oxide (NO) donor or anti-oxidant exhibited significantly increased migration distances compared with high glucose exposed MS1 both at 24 h and 48 h (Fig. 5A,B). Additionally, we performed migration assay based on a Transwell migration system to further evaluate the potential protective role of insulin, L-arginine and β-mercaptoethanol against glucose toxicity (Fig. 5C). After 24 h of incubation in the presence of glucose toxicity on the inserts, MS1 exhibited an approximately 70% decrease in migration over control group. And the migration distances of insulin-treated, L-arginine- and β-mercaptoethanol-supplemented MS1 were shown to have increased by greater than 280%, 265% and 300% respectively compared to HG group (Fig. 5D,E). Taken together, migration analysis supports the hypothesis that glucose toxicity suppresses function of iECs.

## Discussion

Hypertension is a systemic chronic disease involving multiple systems and causing organs and tissues damage. It has been demonstrated that microcirculation supports the adequate blood flow perfusion to local organs and tissues and maintains blood pressure within a normal range^13^. The microcirculatory system has developed mechanisms that function to couple microvascular oscillations (vasomotion) with physiological metabolic activity demands^14^. Disturbed microvascular blood flow and dysfunctional microvascular vasomotion are two critical factors that induce hemorheological disorders in the microcirculation^15^. The prevalence of hyperglycemia or diabetes in hypertensive population is approximately 32% reported recently in a cross-sectional survey^16^. The microcirculatory mechanisms in regards to the phenomenon are not fully clarified yet. In a study reported by Anna Olverling *et al*.^17^, local pancreatic renin-angiotensin system (RAS) controls pancreatic islet blood flow and insulin secretion and thereby may affect blood glucose level. Furthermore, several pancreatic islet RAS signaling pathways seem to be up-regulated by hypertension^18,19^. Therefore, it is possible that the common co-morbidity hyperglycemia in hypertensive individuals has some impact on pancreatic islet microcirculation. In the current study, we demonstrated for the first time that the functional status of pancreatic islet microvascular vasomotion was impaired in SHRs and that hemodynamic disturbance of pancreatic islet microvascular vasomotion might be attributed to elevated blood glucose level.

A characteristic of microvascular vasomotion is the blood perfusion level in microvessels, which is reflected by average blood perfusion. In patients with essential hypertension, the elevated blood press was associated with reduced maximal blood flow^20^. In the present study, we showed that SHRs exhibited decreased average blood perfusion and poor blood flow distribution pattern in pancreatic islet compared with its normotensive WKYs. Concomitant changes in microvessels density in hypertension may be associated with decreased blood perfusion and disturbance in blood glucose level. Microvascular remodeling and rarefaction would reduce the average blood perfusion and impede the exchange of glucose and other nutrients with organs and tissues^21,22^. Our previous data revealed that the density of microvessels was significantly decreased in SHRs compared with WKYs, and considering the current data, we speculate that microvascular rarefaction may be one of the explanations for the decreased microvascular blood perfusion. Additionally, regulating blood flow perfusion requires coordinated vasodilation and vasoconstriction (microvascular vasomotion) among microvessels in the microcirculation^23^. High blood pressure and hemodynamic forces (especially fluid shear stress in pancreatic islet microcirculation) may be other possible explanations of decreased blood perfusion of pancreatic islet microvascular vasomotion that cannot meet the demands required to respond to blood glucose fluctuations. These observations indicate that there is a microcirculatory inadequate blood perfusion of pancreatic islet in hypertensive individuals.

Hemorheological disturbance generally appears in the form of red blood cell aggregation and subsequent decrease of blood flow velocity in microvascular lumina. In the current study, we demonstrated that homeostasis of pancreatic islet microvascular vasomotion was disrupted in SHRs, however, the relative velocity was comparable between SHRs and WKYs. The changes in relative blood velocity remains controversial in hypertension. It has been reported that organ- and tissue- specific arteries exhibit different blood flow velocities when blood pressure elevated. The blood flow velocities of the ophthalmic artery and the central retinal artery were decreased, which might be related with increased peripheral resistance in hypertensive patients^24^. Blood flow velocity of the middle cerebral artery in hypertension was also decreased^25^. However, Perret RS *et al*. reported that the peak blood velocity was increased and significantly correlated with systolic blood pressure in subjects with elevated blood pressure^26^. A compensatory role through which high blood pressure accelerates relative blood velocity could be one of the explanations. Whether increased or decreased blood velocity and changing erythrocyte momentum in the microcirculation may prompt impaired microcirculation are still need to be demonstrated.

Considering glucose toxicity is closely related with damaged functional status of pancreatic islet microvascular vasomotion and iECs are the basic functional unit for pancreatic islet microvascular vasomotion, we hypothesize that glucose toxicity may be involved in iECs dysfunction. NO is a well-known major regulator of vascular function, which is produced by eNOS. p-eNOS^ser117^ has been shown to increase NO production from eNOS^27^ and meanwhile enhance eNOS activity and alter the sensitivity of the enzyme to calcium ion^28^. Our present study showed that the expressions of eNOS and p-eNOS^ser1177^ was significantly decreased in high glucose exposed MS1 compared with euglycemia MS1. In contrast, both expression levels of eNOS and p-eNOS^ser117^ were increased in insulin-treated MS1. The mechanism by which insulin protects iECs from high glucose injury is not entirely understood yet. It is established that eNOS expression in endothelial cell is subject to regulation by pathophysiological factors including insulin and cytokines *etc*.^29^. Insulin enhances eNOS mRNA and protein by activation of phosphatidylinositol 3′ kinase-dependent pathway^30^. And there would be a significant decrease in eNOS mRNA expression in insulin receptor knockout mouse^31^. Consistent with the role for insulin to up-regulate eNOS transcription, it is demonstrated that mice with hyperinsulinemia have increased eNOS mRNA and NO production. Furthermore, insulin can regulate eNOS activity through a calcium ion-independent way^32^. Together with our data, it can be possible that insulin may act as microcirculatory beneficial regulator for microvascular endothelial cells partially through eNOS related pathways.

Additionally, dimerization is required for eNOS catalytic function. eNOS uncoupling is generally a mechanism that leads to endothelial cell dysfunction with or without changes in eNOS expression. In our experiment, there was a clear evidence of an increased ratio of eNOS monomer to eNOS dimer in high glucose exposed group and we speculated that the increased eNOS monomer might be a cause of eNOS uncoupling and iECs dysfunction. Consisting with early study^33^, we confirmed that SHRs exhibited a higher plasma nitrate level compared with WKYs. An increased release of NO from protein-bound dinitrosyl nonheme iron complexes in microvasculature may be one of the possible explanations^34^. On the other hand, the nitrite levels in high glucose exposed MS1 was increased as well and was decreased in insulin treated and β-mercaptoethanol supplemented group, which was to be expected as anti-hyperglycemia or anti-oxidant effects.

Hyperglycemia-induced oxidative stress has been implicated in pathological condition in which nicotinamide adenine dinucleotide phosphate oxidase is a major source of reactive oxygen species (ROS) generation^35^. Meanwhile, ROS accumulated in cells after high glucose incubation also has been reported^36^. And the endothelial barrier dysfunction is documented which is associated with ROS generated primarily by endothelial cells. Our results demonstrated that β-mercaptoethanol supplementation reduced the increased MDA level of high glucose exposed MS1, which may be explained by the inhibition of oxidative stress derived from glucose toxicity. Additionally, L-arginine is required for NO synthesis by eNOS^37^. To confirm the role of eNOS in oxidative stress process, we supplemented L-arginine and found a decreased MDA level in high glucose exposed MS1. These data suggests glucose toxicity induced oxidative stress is of importance in iECs dysfunction from the microcirculatory view.

Endothelial cells function generally identified by proliferation, migration and capillary-like tube formation capacity and permeability^38^. And it is proved that well-behaved selective characteristic of microvascular endothelial cells is crucial for the physiological functions including microvessels tone^39^. Supporting the importance of suited glucose concentration in maintaining iECs function, our results revealed that barrier function of MS1 in high glucose presented medium was breakdown. In addition, high glucose exposed MS1 failed to connect to each other and the tube formation and angiogenic potentials were inhibited. The capacity to form capillary like structure was decreased approximately 50% in high glucose exposed MS1. And glucose toxicity inhibited migration capacity of MS1 in comparison with control iECs, which indicated that glucose toxicity had a significant effect on iECs function. In contrast, notable improvements of barrier function, migration and tube formation capacities were observed in insulin-treated, L-arginine- and β-mercaptoethanol-supplemented iECs respectively. The proposed mechanism of the iECs dysfunction induced by glucose toxicity are similar to the effects of pathological stimuli such as oxidative stress. Moreover, Hatakeyama *et al*. reported that eNOS and eNOS-derived NO were important in signaling pathway for regulation of permeability^40^. Meanwhile, by using eNOS-deficient mice, it is proved that eNOS plays a predominant role in angiogenesis^41^. Taken together, these lines of findings confirm that glucose toxicity has an iECs functional disadvantages in microcirculatory level. Elevated glucose level in SHRs plays a role in iECs dysfunction and might be involved in the pathologic impairment of pancreatic islet microvascular vasomotion. And due to anti-hyperglycemic and anti-oxidative effects, partly through mechanism involving regulation of eNOS and p-eNOS^ser1177^, insulin, L-arginine and β-mercaptoethanol supplement have a functional benefit of iECs.

As microvascular vasomotion dysfunction may promote the progression of diseases, strategies to uncover the intertwined relationship in hypertension-related blood glucose elevation and to ameliorate the microvascular vasomotion dysfunction may be theoretically beneficial for improving hypertension-associated symptoms. Several studies have mentioned that microvascular vasomotion may be considered as a new therapeutic target in the pathophysiology and treatment of hypertension^42^ and its concomitant complications^43^. Furthermore, insulin-induced vasodilatation *in vivo* has been attributed to the activation of eNOS^30^. Our previously data demonstrated that insulin administration could restore the bio-rhythmic of microvascular vasomotion *in vivo* ^12^. Therefore, we propose that the stable blood flow perfusion in the entire body or in loco-regional organs and tissues may become an approach for the clinical treatment of hypertension. Several drugs targeting microcirculation have been shown to be beneficial for long-term management of hypertension. Evidence indicates that calcium antagonists are beneficial for microvascular endothelial cells function by improving endothelial cell-dependent microvascular vasomotion in stenotic vessels of hypertensive individuals^44^. Improving vasomotion via cholesterol-lowering and antioxidant therapies may reduce the incidence of adverse coronary events in patients with hypertension and coronary artery disease^45^. Excessive vasoconstriction can increase blood pressure, hence blocking receptors such as transient receptor potential channels and their signal pathways^46^ may reduce the elevated blood pressure. Moreover, due to microvascular endothelial cells are the primary type of cell that exerts microvascular vasomotion^47^, it can be speculated that improving microvascular endothelial function should also be considered, such as angiotensin-converting enzyme inhibitors^48^ and angiotensin receptor blockers^49^.

In summary, the present study revealed the impaired microcirculatory blood perfusion pattern and the pathological changed pancreatic islet microvascular vasomotion in SHRs, which was negatively correlated with blood glucose level. Treatment with insulin *in vivo* or administration with L-arginine or β-mercaptoethanol *in vitro* improved functional status of pancreatic islet microvascular vasomotion and iECs function probably through the regulation of eNOS, p-eNOS^ser1177^ and the protective effects against oxidative stress derived from glucose toxicity. Thus, our findings indicated that iECs might be a novel therapeutic target for pancreatic islet microvascular vasomotion dysfunction in hypertension.

## Materials and Methods

### Animals

The study was approved by the Institute of Microcirculation Animal Ethics Committee (IMAEC) at the Peking Union Medical College (PUMC). All experiments in the current study were performed in accordance with relevant guidelines and regulations. Eight-week-old specific pathogen-free male SHRs and WKYs were purchased from the Institute of Laboratory Animal Science [Chinese Academy of Medical Science (CAMS) & PUMC, Beijing, China]. Regular diet was obtained from the Institute of Laboratory Animal Science (CAMS). Meanwhile, another group of eight-week-old SHRs were administrated 0.5 IU/day insulin subcutaneously for one week to maintain blood glucose in normal range (SHR-Ts group). Rats (*n* = 6 per group) were provided a standard laboratory diet and free access to tap water and were housed in a room with controlled temperature (22 ± 1 °C) and humidity (65 ~ 70%) under a 12:12 h light: dark cycle. All rats were weighed, and blood glucose baselines were obtained from the tail vein using One Touch UltraEasy^®^ glucometer (Lifescan).

### Blood pressure

Rats were anesthetized with 3% pentobarbital sodium and placed in a lateral decubitus position. A 10-mm midline longitudinal incision was made over the thyroid bone. The common carotid artery and external carotid artery were exposed and dissected. A permanent ligature was placed on distal segment while a temporary 6–0 prolene ligature was placed on the proximal segment to obtain a flow-free segment of common carotid artery. A micro-catheter was introduced into the carotid artery and advanced in retrograde fashion toward the carotid bifurcation. The catheter was then secured and rotated so that the micro-catheter tip was oriented cephalad in the internal carotid artery. The micro-catheter was attached to a combination pressure transducer and transmitter. Blood pressure was measured by MP150 data acquisition and analysis systems with AcqKnowledge software (BIOPAC). Systolic blood pressure (SBP), diastolic blood pressure (DBP) and mean arterial pressure (MAP) were recorded.

### Intraperitoneal glucose tolerance test and insulin tolerance test

To evaluate the function of pancreatic islet β cells and the ability to regulate blood glucose level, an intraperitoneal glucose tolerance test was performed. Briefly, rats were fasted overnight for 16 h, and an intraperitoneal injection of D-glucose solution (2 g/kg body mass) was administrated. Blood samples were collected from the tail vain immediately before and at 15, 30, 60 and 120 min after glucose injection for blood glucose measurements. The area under the curve (AUC) for glucose (AUC_glucose_) was calculated by the trapezoidal method using GraphPad Prism 6.0 (GraphPad). The concentration of blood glucose was analyzed with a glucometer and disposable tips (Lifescan) mentioned above. An insulin tolerance test was also performed in the two groups of rats. 1 IU/kg body weight insulin i.p. was given to non-fasted rats. A blood sample was then obtained via tail nick, and blood glucose levels were measured at 15, 30, and 60 min. The AUC for insulin (AUC_insulin_) was calculated.

### Assessment of pancreatic islet microvascular vasomotion

A dual-channel laser Doppler monitoring instrument and Moor software (Moor) were used to evaluate pancreatic islet microvascular vasomotion as previously described^50^. Briefly, after anesthetizing with 3% pentobarbital sodium, rats were placed in a supine position and incised around upper abdomen to expose the pancreas. The electrode was advanced to within 1 mm of the pancreas to collect data of pancreatic islet microcirculation. The probes were repositioned after each measure to avoid additive effects and localized exhaustion of contraction and relaxation ability. Changes in blood perfusion unit (PU) of pancreatic islet microvascular vasomotion parameters were evaluated. The frequency was defined as the number of microvascular vasomotion waves per minute. The amplitude was calculated as the difference between the maximum PU and the minimum PU (∆PU). Data derived from a non-reflective plate were used as a negative control to validate the evaluation method.

### Cell culture

Islet microvascular endothelial cells (iECs) MS1 were obtained from American Type Culture Collection (ATCC) and cultured. MS1 were grown in Dulbecco’s Modified Eagle’s Medium (DMEM, Gibco), supplemented with 10% FBS (Bovogen), 5.6 mmol/L glucose, 4 mmol/L L-glutamine, 100 U/mL penicillin and 100 *μ*g/mL streptomycin under standard conditions (5% CO_2_ at 37 °C). After cells were confluent, MS1 cells were divided into 5.6 mM glucose treated group (control group), 35 mM glucose treat group (HG group), 35 mM glucose plus 10^−8^ M insulin treated group (HG + I group), 35 mM glucose plus 0.5 mM L-arginine treated group (HG + R group) and 35 mM glucose plus 100 μM β-mercaptoethanol treated group (HG + βME group).

### Immunohistochemistry

An immunolabeling horseradish peroxidase (HRP) system were used for visualization of insulin and eNOS levels. To determine the expression of insulin in pancreatic islets tissue, deparaffinized sections were treated with 3% hydrogen peroxide for 30 min and blocked with 5% bovine serum albumin (BSA; TBD Science Technology). And then sections were incubated with anti-insulin antibody (1:50, Santa Cruz) overnight at 4 °C. After washing with phosphate buffered saline (PBS, pH 7.4), sections were incubated with HRP polymer conjugated secondary antibody for 1 h. After washing step, sections were reacted with 3,3′-diaminobenzidine (DAB) for 10 min and counter-stained with Mayer’s hematoxylin. Positive staining in pancreatic islets was analyzed in five different high-power fields using a Leica DFC450 (Leica). Quantitative analysis was accomplished using Image Pro Plus 6.0 (Media Cybernetics). To visualize eNOS expressed on iECs, MS1 were seeded on EZ Slide (Millipore) at 1 × 10^4^ per well and were fixed in 4% paraformaldehyde for 30 min, followed by permeabilization for 45 min in 0.25% Triton X-100. And sequentially treated with 2.5% normal horse serum for 20 min to block nonspecific labeling. The anti-eNOS antibody (1; 50, Abcam) were incubated for 90 min, followed by incubating in Amplifier antibody for 15 min. After washing step, cells were incubate for 30 min with ImmPRESS^TM^ Excel Reagent for 30 min, and incubated in DAB working solution for 15 min. After rinsing in tap water and mounting, the intensity was observed. A reference image was taken in a blank area without cells. The intensity was defined as Ic - Ig, where Ic is the transmitted light intensity of labeled eNOS on MS1 and Ig is the transmitted light intensity of glass.

### Enzyme linked immunosorbent assay (ELISA)

Serum insulin level and plasma MDA level were determined by using competitive ELISA kits (BlueGene). Briefly, 100 μL sera samples or cell culture supernatants and gradient standards were added to appropriate well in duplicate. 50 μL conjugation solution was then added to each well, mixed and incubated for 1 h at 37 °C. After washing, 50 μL of substrate A and substrate B were added to each well and subsequently incubated for 15 min at room temperature. The reaction was stopped by adding 50 μL stop solution and absorbance was measured by microplate reader (Thermo Fisher Scientific) at 450 nm.

### Measurement of total Nitrite/Nitrate levels

Plasma samples of SHRs, WKYs and SHR-Ts and cell culture supernatants of different groups were collected and prepared. Nitrite/Nitrate concentrations were assayed with a Nitrite/Nitrate immunoassay kit (R&D System) using Griess reagents following the instructions. Absorbance was measured by microplate reader (Thermo Fisher Scientific) at 540 nm. The results were expressed in μmol/L.

### Western blot analysis

To detect the expressions of eNOS and p-eNOS^ser1177^ in iECs, MS1 cells in control, HG, HG + I, HG + R and HG + βME groups were suspended in lysis buffer containing a protease inhibitor cocktail (Pierce Biotechnology) and phosphatase inhibitors (Roche). All cellular samples were sonicated for 10 s. The concentration of proteins were quantified by a BCA assay kit (CWBiotech) according to the manufacturer’s instructions. Proteins were heated at 95 °C for 10 min in loading buffer. Equal amount of proteins (20 μg/sample) were loaded and separated by 10% sodium dodecyl sulfate-polyacrylamide gel electrophoresis (SDS-PAGE) and then transferred onto polyvinylidene difluoride membranes using iBlot^TM^2 gel transfer system (Invitrogen). Non-specific bindings were blocked with 5% (v/v) non-fat dry milk in phosphate-buffered saline (PBS) at room temperature for 1 h. The membrane was then incubated with primary antibodies against eNOS and p-eNOS^ser1177^ (1: 500 dilution; Invitrogen) at 4 °C overnight. After washing, membranes were incubated with a peroxidase-conjugated goat anti-rabbit IgG secondary antibody (1: 5000 dilution; CWBiotech) for 1 h. Bands were visualized by enhanced chemiluminescence (Pierce Biotechnology). Protein bands were quantified by densitometry using ImageJ (National Institutes of Health). The expression of protein levels were normalized against β-actin.

### Low-temperature SDS-PAGE (LT-PAGE)

LT-PAGE was performed to detect eNOS monomer and dimer in different groups^51^. Proteins were incubated in 1 × Laemmli buffer without 2-mercaptoethanol at 37 °C for 5 min and then subjected to SDS-PAGE. Gel and buffers were equilibrated at 4 °C before electrophoresis and buffer tank was placed in an ice bath during electrophoresis to maintain the low temperature (less than 15 °C). Gels were then transferred as routine Western blot mentioned above.

### Islet microvascular endothelial cells permeability assays

The permeability of islet endothelial cells was determined by measuring the passage of FITC-dextran (2 mg/ml, molecular mass: 40 kDa; Sigma) through MS1 monolayer. MS1 at full confluence was grouped and treated as mentioned above for 24 h. FITC-conjugated dextran was added to the upper chamber of the Transwell system (Costar). 100 μL aliquots were collected from the lower chamber at 0, 5, 15, 30 and 60 min respectively. The fluorescence leaked to the lower chamber was measured using a fluorescence multiwall plate reader (Bio-Tek). The FITC-dextran levels were calculated and normalized against with the fluorescence value at baseline.

### Tube formation

To determine whether glucose toxicity plays a role in angiogenesis capacity of iECs, a tube formation assay was performed *in vitro* as described previously^52^. MS1 at full confluence was grouped and treated as previous. Matrigel (BD) was spread evenly over each well (50 μL) of a 96-well plate. The plate was incubated for 30 min at 37 °C to allow the Matrigel to solidification. Pre-treated MS1 cells were seeded at 5 × 10^4^ per well and grown in 100 μL DMEM supplemented with 5% FBS for 2 h in a humidified 37 °C, 5% CO_2_ incubator. Tube formation was observed and five representative fields from each well were captured and analyzed using WimTube Release 4.0 in Wimasis Image Analysis System (Wimasis, https://www.wimasis.com/en/products/13/WimTube).

### Scratch wound healing assay

Scratch wound healing assay was performed *in vitro* after a monolayer MS1 were wounded. The confluent MS1 monolayer was scraped with a 200 μL pipette tip to create a straight-lined wound parallel to the flow direction. The wounded monolayers were washed with the growth medium to remove the debris and exposed to conditioned medium. Images of the cell monolayer around the wounds were obtained and wound areas were visualized by staining with 0.1% crystal violet for 20 min. Wound closure distances in the microscopic images were quantified using the ImageJ 1.42q program (NIH), and were calculated as: Wound distance (μm) = A0 − At, where A0 is the original wound distance and At is the distance measured at a specified time after scratching.

### Migration assay

A quantitative haptotactic migration assay was performed as described previously^53^. In brief, migration of MS1 was assayed in Transwell chambers with 6.5 mm diameter polycarbonate membrane filters containing 8 μm pore size (Costar). 5 × 10^4^ MS1 were seeded and cultured in the upper chamber and the lower chamber was filled with 600 μL culture medium with 5% fetal bovine serum (Bovogen). MS1 were grouped as mentioned above and were incubated at 37 °C for 24 h. Un-migrated cells from the upper surface of the membrane were removed with a cotton swab. And then filters were fixed in methanol for 10 min and stained by 0.1% crystal violet for 20 min. After washing, the average number of migrated MS1 in five randomly chosen fields was counted and the mean number was calculated.

### Statistical analyses

The statistical analyses were performed using SPSS 17.0 for Windows (IBM). All values in the tables and figures were presented as the mean ± standard error of the mean (S.E.M.) of *n* independent experiments unless indicated. Data were subjected to *t* tests (between two groups). AUCs were calculated by the trapezoidal method using GraphPad Prism 6.0 (GraphPad). The correlations between blood glucose level and pancreatic islet microvascular vasomotion parameters were analyzed with Pearson’s correlation coefficients. Probabilities of 0.05 or less were considered to be statistically significant.

### Data Availability

The datasets generated and analyzed during the current study are available from the corresponding authors on reasonable request.

## Electronic supplementary material

Supplementary Information
