# Supplementary material for: Functional status of microvascular vasomotion is impaired in spontaneously hypertensive rat
Source: Sci Rep. 2017 Dec 6;7:17080. doi: 10.1038/s41598-017-17013-w (PMC5719042; doi:10.1038/s41598-017-17013-w)
Supplement: Supplementary file 1 — Supplementary Information [file 41598_2017_17013_MOESM1_ESM.pdf]

Supplementary Information

Functional status of microvascular vasomotion is impaired in spontaneously hypertensive rat

Mingming LIU[1], Xiaoyan ZHANG[1], Bing WANG[1], Qingbin WU[1], Bingwei LI[1], Ailing LI[1,*], Honggang ZHANG[1,*] & Ruijuan XIU[1]

[1]Institute of Microcirculation, Key Laboratory of Microcirculation, Ministry of Health, Chinese Academy of Medical Sciences & Peking Union Medical College, Beijing 100005, China

*** Corresponding author A**

Prof. Ailing LI
Institute of Microcirculation
Chinese Academy of Medical Sciences & Peking Union Medical College
No.5 Dong Dan San Tiao, Dongcheng District, Beijing, 100005, China
Phone: +86 10 65126407
Fax: +86 10 65251957
Email address: lialwork@imc.cams.cn

*** Corresponding author B**

Prof. Honggang ZHANG
Institute of Microcirculation
Chinese Academy of Medical Sciences & Peking Union Medical College
No.5 Dong Dan San Tiao, Dongcheng District, Beijing, 100005, China
Phone: +86 10 65126407
Fax: +86 10 65251957
Email address: zhanghg1966126@163.com

Supplementary Data

Number of Supplementary Figures: 1
Number of Supplementary Tables: 1

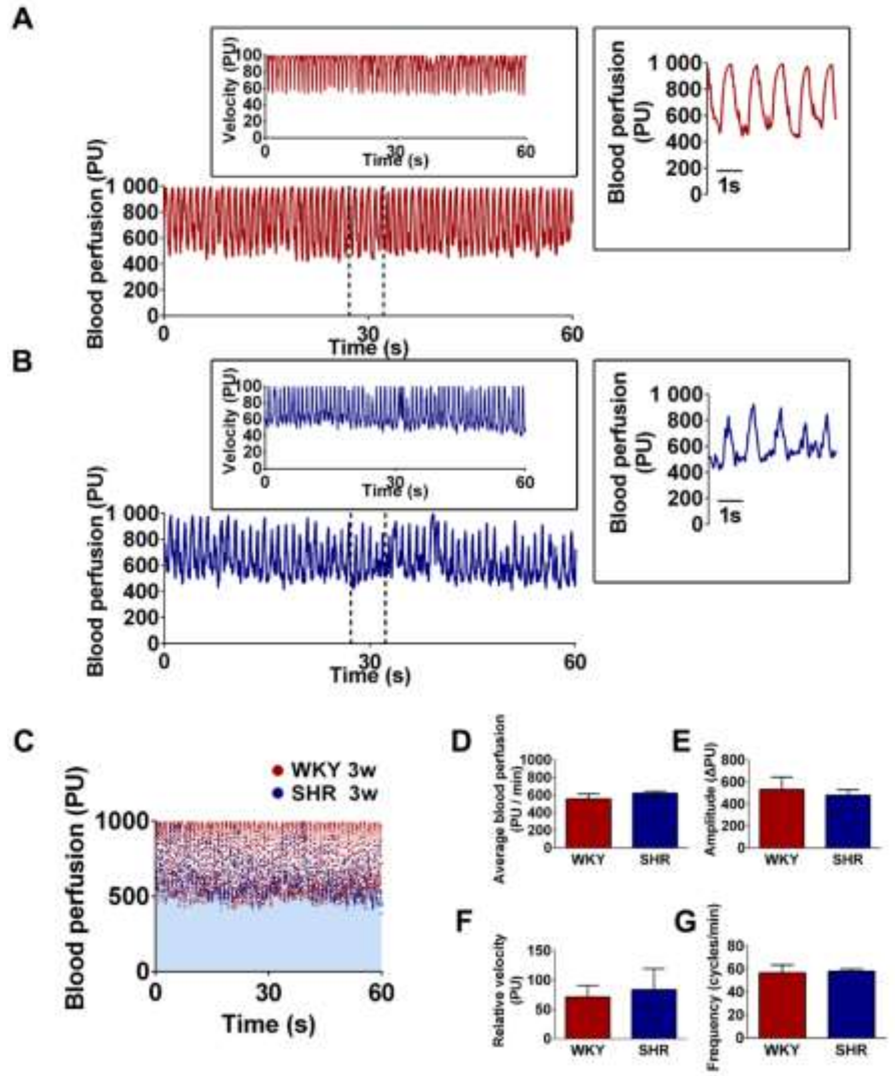

Supplementary Figure S1. Functional status of pancreatic islet microvascular vasomotion in three-week-old SHRs and WKYs. (A) Functional status of pancreatic islet microvascular vasomotion in three-week-old WKYs. **(B)** Functional status of pancreatic islet microvascular vasomotion in three-week-old SHRs. The dynamic velocity of microvascular blood flow was shown in the rectangular insert. The microvascular blood perfusion between the dashed lines (5 s) was extracted in the square insert. The red line represents microvascular blood perfusion data of three-week-old WKYs; the blue line represents perfusion data of three-week-old SHRs; PU, perfusion unit. **(C)** Distribution patterns of pancreatic islet microvascular blood perfusion in three-week-old WKYs and SHRs. The distribution pattern was analyzed using microvascular blood perfusion data derived from laser Doppler monitoring. Three-week-old WKYs and SHRs showed similar blood perfusion pattern. Red dots, microvascular blood perfusion of three-week-old WKYs. Blue dots, microvascular blood perfusion of three-week-old SHRs. PU, perfusion unit. **(D-G)** Quantification analysis of functional status of pancreatic islet microvascular vasomotion in three-week-old WKYs and SHRs. No significantly differences were found in pancreatic islet microvascular vasomotion parameters (including average blood perfusion, amplitude, relative velocity and frequency) between three-week-old WKYs and SHRs.

Supplementary Table S1 Body weight, blood glucose and blood pressure of three-week-old WKYs and SHRs

Groups	WKYs (*n* = 6)	SHRs (*n* = 6)
Age (weeks)	3	3
Gender (male / female)	6/0	6/0
Body weight (g)	39.5 ± 1.3	42.7 ± 1.5
FPG (mg/dL)	65.5 ± 4.4	62.4 ± 2.2
Blood pressure (mmHg)		
SBP	109.0 ± 1.5	128.0 ± 1.7 **
DBP	74.7 ± 6.0	89.7 ± 0.3
MAP	94.7 ± 6.1	107.3 ± 1.5

Unless indicated otherwise, numbers were expressed as the mean ± S.E.M. (*n* = 6 in each group). FPG, fasting plasma glucose; SBP, systolic blood pressure; DBP, diastolic blood pressure; MAP, mean arterial pressure.**, $P < 0.01$ compared with WKYs.

Full-length blot of representative Western Blot images of Figure 3C

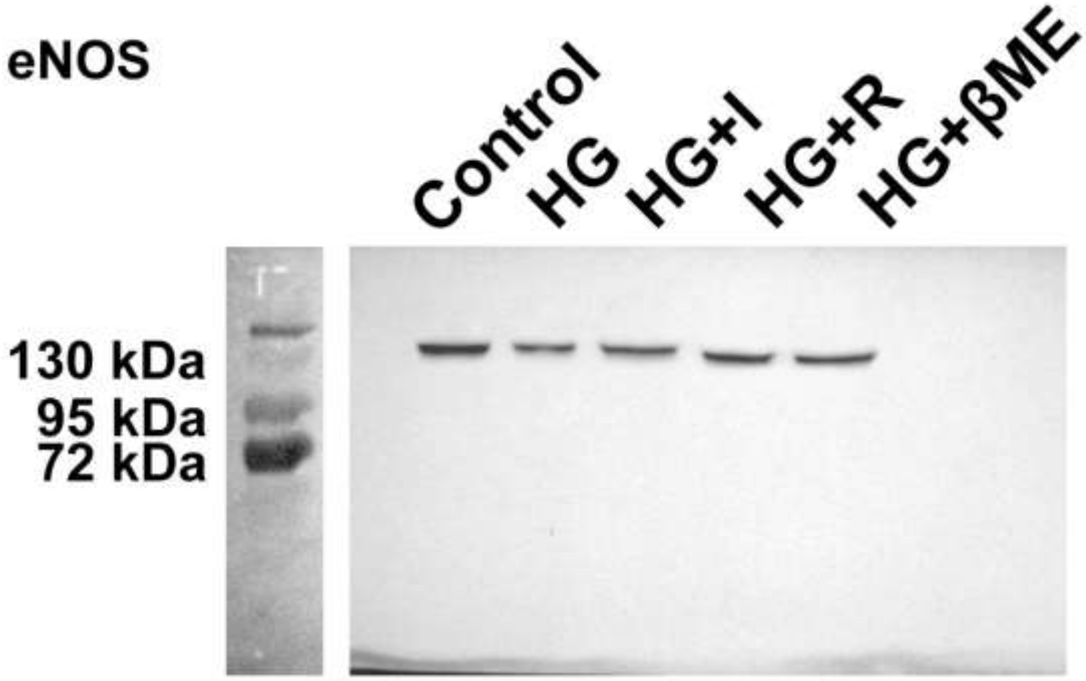

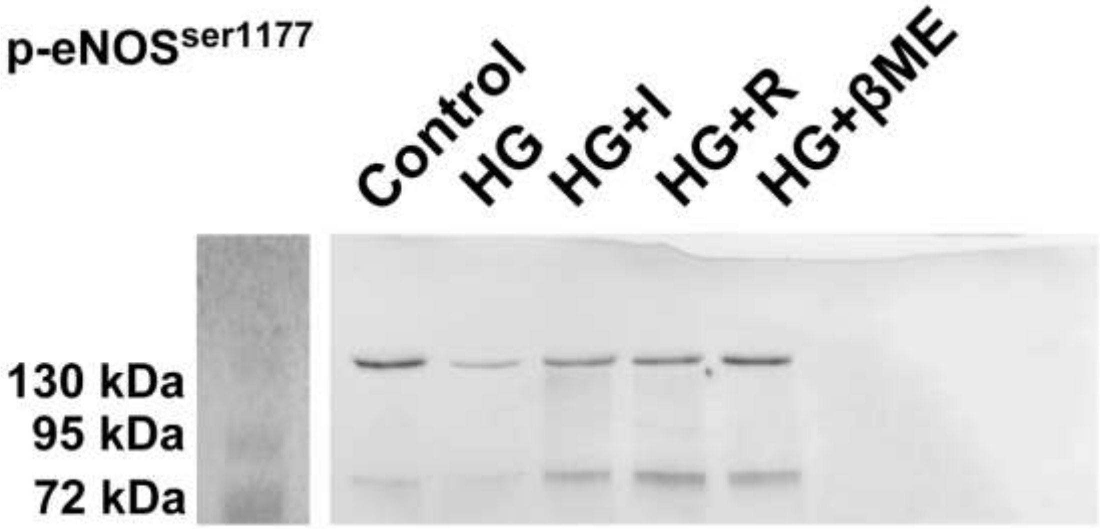

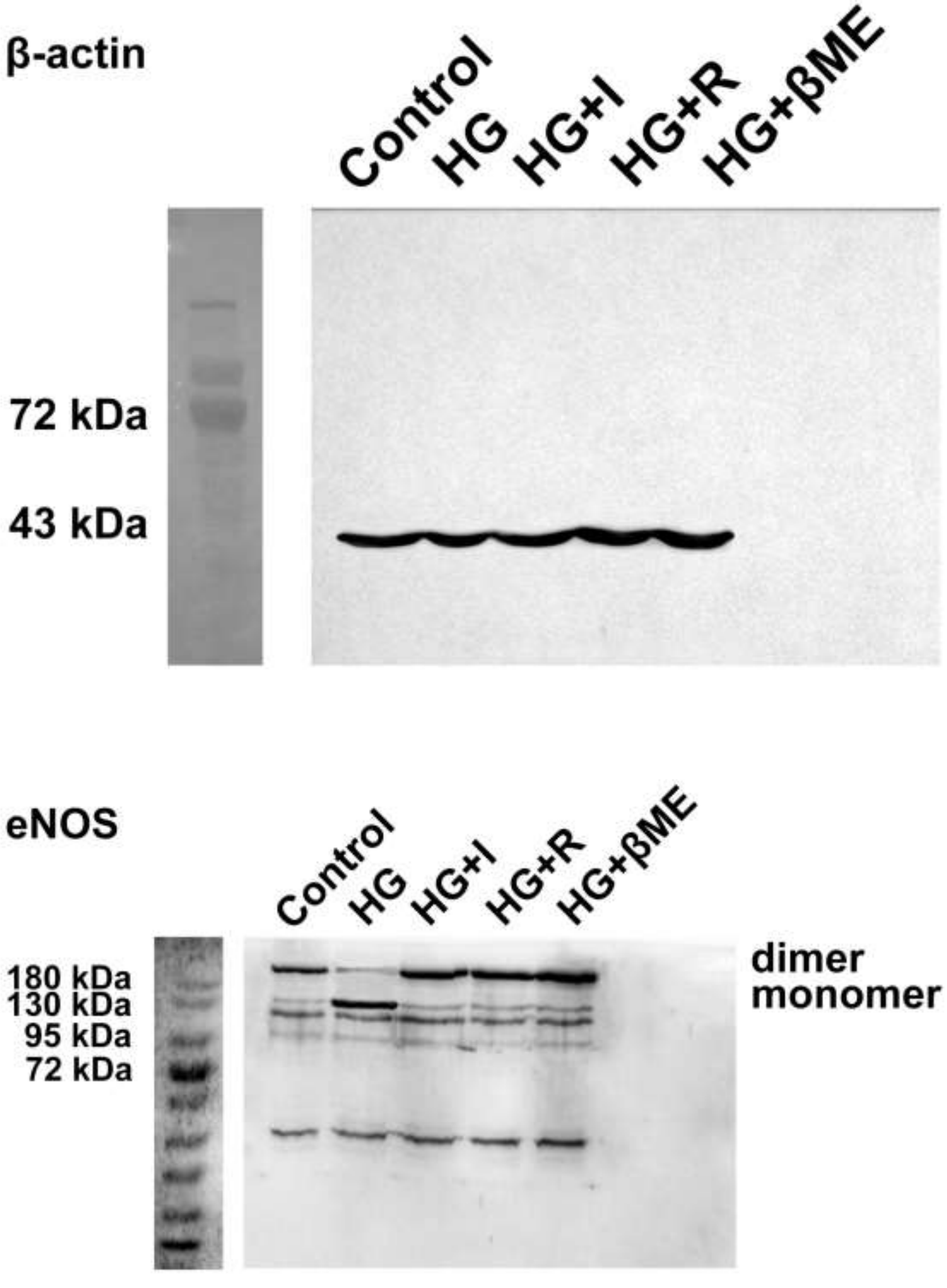
β-actin
Control
HG
HG+I
HG+R
HG+βME
72 kDa
43 kDa
eNOS
Control
HG
HG+I
HG+R
HG+βME
180 kDa
130 kDa
95 kDa
72 kDa
dimer
monomer
